# Rate-Associated Differences in Within-Session Motor-Inhibitory Performance: Exploratory Role of Affective Temperament

**DOI:** 10.3390/life16081368

**Published:** 2026-08-19

**Authors:** Luca Puce, Gabriele Pretelli, Carlo Trompetto

**Affiliations:** 1 Department of Neuroscience, Rehabilitation, Ophthalmology, Genetics, Maternal and Child Health (DINOGMI), University of Genoa, 16132 Genoa, Italy; gabrielepretelli2001@gmail.com (G.P.); ctrompetto@neurologia.unige.it (C.T.); 2 IRCCS Azienda Ospedaliera Metropolitana, 16132 Genoa, Italy

**Keywords:** cognitive-motor inhibition, response inhibition, motor control, affective temperament, stimulus rate

## Abstract

Response inhibition is often summarized as a single individual score, although performance may change within a session as temporal demand and repeated exposure vary. We examined whether change in No-Go motor performance differed across stimulus-rate conditions and was associated with affective temperament in 53 physically active participants, predominantly young adults. Participants completed three blocks of an elbow-based Go/No-Go task; four 50-stimulus series were administered at 40, 50, 60, and 70 bpm in randomized order. The analysis included 636 series-level observations and 6360 No-Go trials, including 3185 commission errors (50.1%). Commission-error occurrence was given primary interpretive priority; conditional error duration was considered complementary, and the pragmatically weighted integrated error index was treated as a secondary composite. Error burden differed across stimulus-rate conditions, and rate-by-block interactions emerged for commission-error occurrence (trial-level GEE; Wald χ^2^(6) = 25.96, *p* < 0.001), conditional error duration (Wald χ^2^(6) = 14.96, *p* = 0.021), and the integrated index (Wald χ^2^(6) = 30.40, *p* < 0.001). Direct contrasts confirmed larger Block-1-to-Block-3 reductions at 50–70 bpm than at 40 bpm. A secondary exploratory participant-level analysis suggested that higher irritable temperament was associated with a more negative Block-3-minus-Block-1 change (β = −0.82, 95% CI −1.39 to −0.26, FDR-adjusted *p* = 0.020), although the corresponding moderation did not survive global correction across all temperament tests. Overall, the data indicate rate-associated differences in within-session No-Go motor performance; temperament-related findings remain exploratory.

## 1. Introduction

Response inhibition refers to the ability to suppress actions that are inappropriate, premature, or no longer aligned with current goals, and it is a core component of executive control [1,2,3]. This ability is essential in everyday and sport-related motor behavior, where individuals must continuously decide whether to initiate, withhold, or adjust an action in response to changing environmental demands. In fast cognitive-motor contexts, inhibitory control is therefore not only a matter of avoiding an incorrect response, but also of regulating movement output under time pressure.

Response inhibition should not be considered a unitary process. Different forms of inhibitory control may be involved depending on whether an action must be withheld before execution, interrupted after initiation, or corrected while it is unfolding. Experimental paradigms differ in the inhibitory processes they emphasize. Go/No-Go tasks mainly assess action restraint, namely the ability to withhold a response before it is executed, whereas stop-signal paradigms emphasize action cancelation, namely the interruption of an already initiated response [4]. Recent neuroimaging evidence further indicates that the neural architecture supporting inhibitory control varies according to task complexity and contextual demands [5]. This distinction is relevant for cognitive-motor tasks in which stimuli are presented rapidly, and participants must continuously update action selection.

Inhibitory performance is unlikely to operate as a fixed trait-like capacity. Evidence from the sport-cognition literature suggests that inhibitory control varies with expertise, task constraints, and environmental demands, although findings remain heterogeneous because of differences in experimental design, task structure, and outcome definition [6,7,8,9]. Temporal urgency may alter evidence accumulation, response thresholds, and speed–accuracy policy during ongoing decisions [10,11]. At the same time, repeated exposure to a task can reduce false alarms and modify neural indices of inhibitory control within a single experimental session [12,13], and response-inhibition training may influence GABA-mediated motor-cortical inhibition [14]. These observations suggest that stimulus rate and within-session repetition may interact: practice-related changes may be smaller when the task is relatively slow or performance is already stable but become more evident under faster stimulus-rate conditions.

A further issue concerns the behavioral interpretation of commission errors. In standard Go/No-Go paradigms, commission-error rate is commonly treated as the main indicator of failed action restraint. However, in a cognitive-motor task involving measurable movement output, an error can be decomposed into at least two complementary components. A participant may frequently release an inappropriate movement but suppress it rapidly, whereas another participant may make fewer errors but allow each erroneous movement to persist for longer. These profiles are not equivalent. Commission-error probability primarily reflects the likelihood that restraint fails, whereas the duration of the erroneous movement after onset may reflect the efficiency of online correction or motor suppression once an inappropriate response has escaped. This distinction is consistent with broader models of stopping, which separate whether an inhibitory process is triggered from how efficiently it suppresses an action [15,16]. Therefore, composite measures of total error burden can be useful, but they should be interpreted together with their components because similar composite values may arise from different combinations of error frequency and error persistence.

Individual differences in affective temperament may also influence performance in demanding cognitive-motor tasks. Affective temperament refers to relatively stable patterns of emotional reactivity, energy, and self-regulation, commonly described across depressive, cyclothymic, hyperthymic, irritable, and anxious profiles. Related work has linked anxiety, emotional regulation, and attentional control to inhibitory efficiency, particularly under demanding or stressful conditions [17,18,19,20,21]. These temperament profiles may therefore shape how individuals adjust to changes in temporal demand across repeated task blocks. Because affective temperament is conceived as a dimensional rather than categorical construct, validated psychometric assessment can characterize individual variation across multiple affective domains and relate these profiles to task-dependent behavioral dynamics. This approach is particularly relevant when performance is expected to vary with changing temporal demands rather than reflect a single stable individual characteristic. However, temperament dimensions are often intercorrelated, and trait-based analyses are vulnerable to multiple testing. Associations between affective temperament and inhibitory performance should therefore be examined using simultaneous adjustment and interpreted cautiously.

The present study examined No-Go motor performance during a modified cognitive-motor Go/No-Go protocol in physically active participants, predominantly young adults. The primary objective was to determine whether within-session change differed across randomized stimulus-rate conditions. Rather than treating inhibitory performance as a single static score, we tested whether repeated task exposure produced different performance trajectories across various stimulus-rate conditions. We expected faster stimulus rates to produce greater error burden and examined whether within-session change differed across stimulus-rate conditions. The secondary objective was to determine whether rate-associated within-session change reflected changes in commission-error probability, conditional error duration, or both. Finally, given the potential influence of affective temperament on performance under changing task demands, we explored whether temperament moderated rate- or block-related change when all dimensions were entered simultaneously in the same model.

## 2. Materials and Methods

### 2.1. Participants

Eligibility required structured training at least three times per week during the preceding six months, an age range of 15–35 years, and the ability to understand task instructions. Exclusion criteria were any medical condition or medication likely to affect cognitive or motor function.

The study protocol was approved by the local Ethics Committee of the University of Genoa, Genoa, Italy (protocol no. 2024.36, 12 April 2024). Written informed consent was obtained before study procedures in accordance with the Declaration of Helsinki [22]. Where applicable, consent procedures for participants younger than 18 years followed the approved ethics protocol.

### 2.2. Affective Temperament

Affective temperament was assessed one day before the motor task using the Brief Temperament Evaluation of Memphis, Pisa, Paris and San Diego (Brief TEMPS-M; modified version). The instrument yields depressive, cyclothymic, hyperthymic, irritable, and anxious scores and has been validated in an Italian sample [23]. Raw subscale scores were retained to preserve their clinical and conceptual interpretation. For multivariable analyses, each score was standardized across participants, and all five temperament dimensions were entered simultaneously to reduce confounding by their intercorrelations. Internal consistency of each seven-item subscale in the present sample was assessed from the item-level responses using Cronbach’s α.

### 2.3. Cognitive-Motor Go/No-Go Task

**Figure 1:**
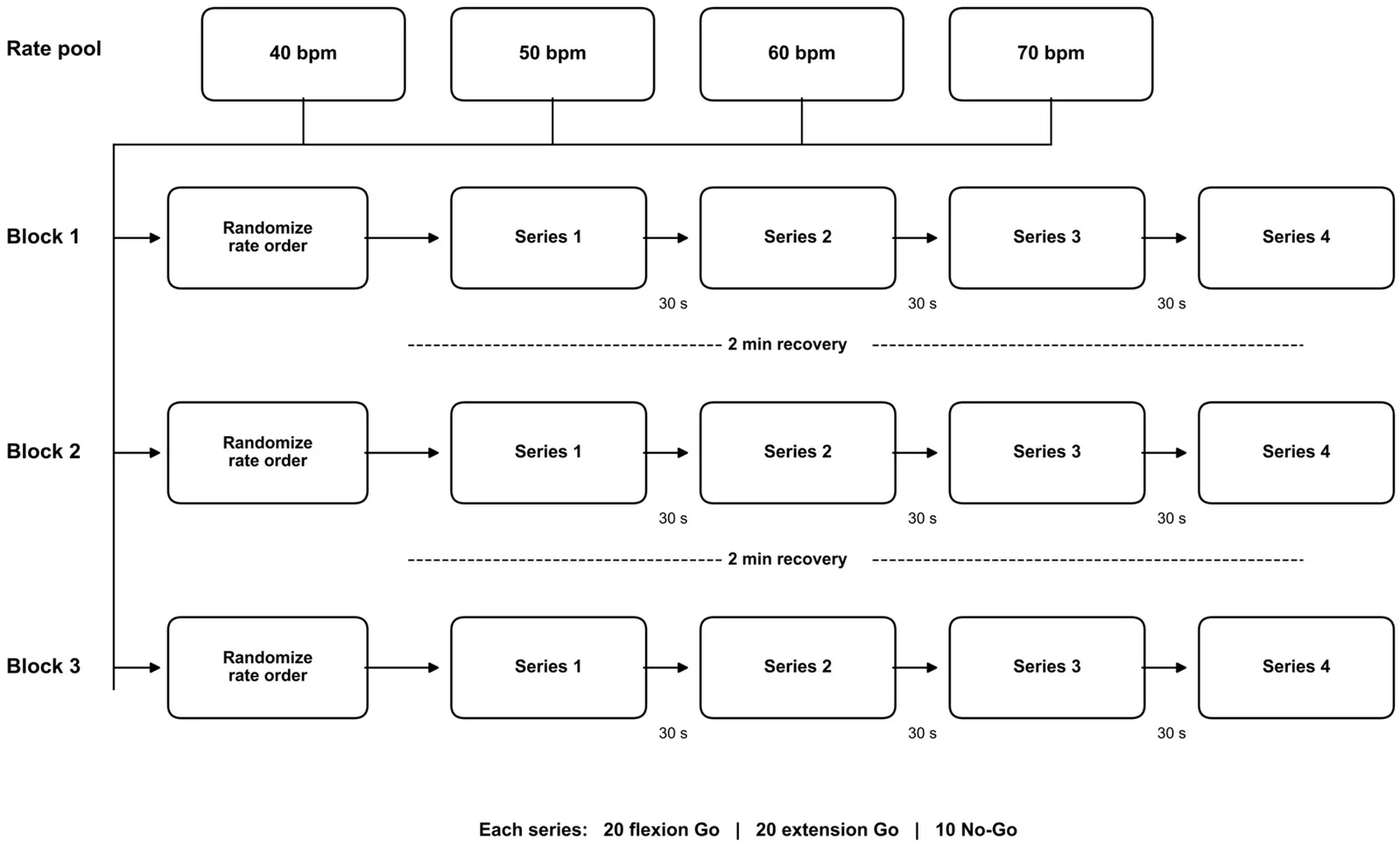
Structure of the cognitive-motor Go/No-Go protocol and stimulus-rate randomization. Each experimental block comprised four 50-stimulus series. The four stimulus-rate conditions (40, 50, 60, and 70 bpm) were administered once per block in randomized order, so that each rate occurred exactly once within each block while its serial position varied according to the randomized order. Each rate was associated with a predefined 50-stimulus sequence containing 20 elbow-flexion Go trials, 20 elbow-extension Go trials, and 10 pseudorandomly interspersed No-Go trials. The same rate-specific sequence was retained across the three blocks and was not counterbalanced across participants. Thirty seconds of rest separated consecutive series, and two minutes of recovery separated blocks.

Participants sat in front of a screen with the dominant upper limb free to perform elbow flexion and extension. An upward arrow required elbow flexion; a downward arrow required elbow extension, and a circle required complete response inhibition. Each 50-stimulus series comprised 20 elbow-flexion Go trials, 20 elbow-extension Go trials, and 10 No-Go trials. When No-Go trials were omitted, the Go stimuli followed a strict alternating flexion–extension pattern. No-Go stimuli were pseudorandomly interspersed, and consecutive No-Go stimuli were not permitted. A predefined 50-stimulus sequence was assigned to each stimulus rate (40, 50, 60, and 70 bpm); the same rate-specific sequence was used for all participants and repeated across the three experimental blocks. Thus, sequence content was fixed within rate and was not counterbalanced across participants, whereas the order of the four rate conditions was randomized within each block. Exact No-Go positions for each rate-specific sequence are provided in Supplementary Table S8. The protocol comprised three consecutive blocks separated by two minutes of recovery, with 30 s of rest between series (Figure 1).

### 2.4. Kinematic Acquisition, IMU Processing, Error Detection, and Outcome Calculation

Movement kinematics were acquired at 500 Hz using four inertial measurement units (Mini Wave X sensors; Cometa, Bareggio, Italy) placed on the dorsum of the hand, forearm, upper arm, and sternum. Acquisition was synchronized with the cognitive-motor task through a trigger generated at each stimulus onset.

**Figure 2:**
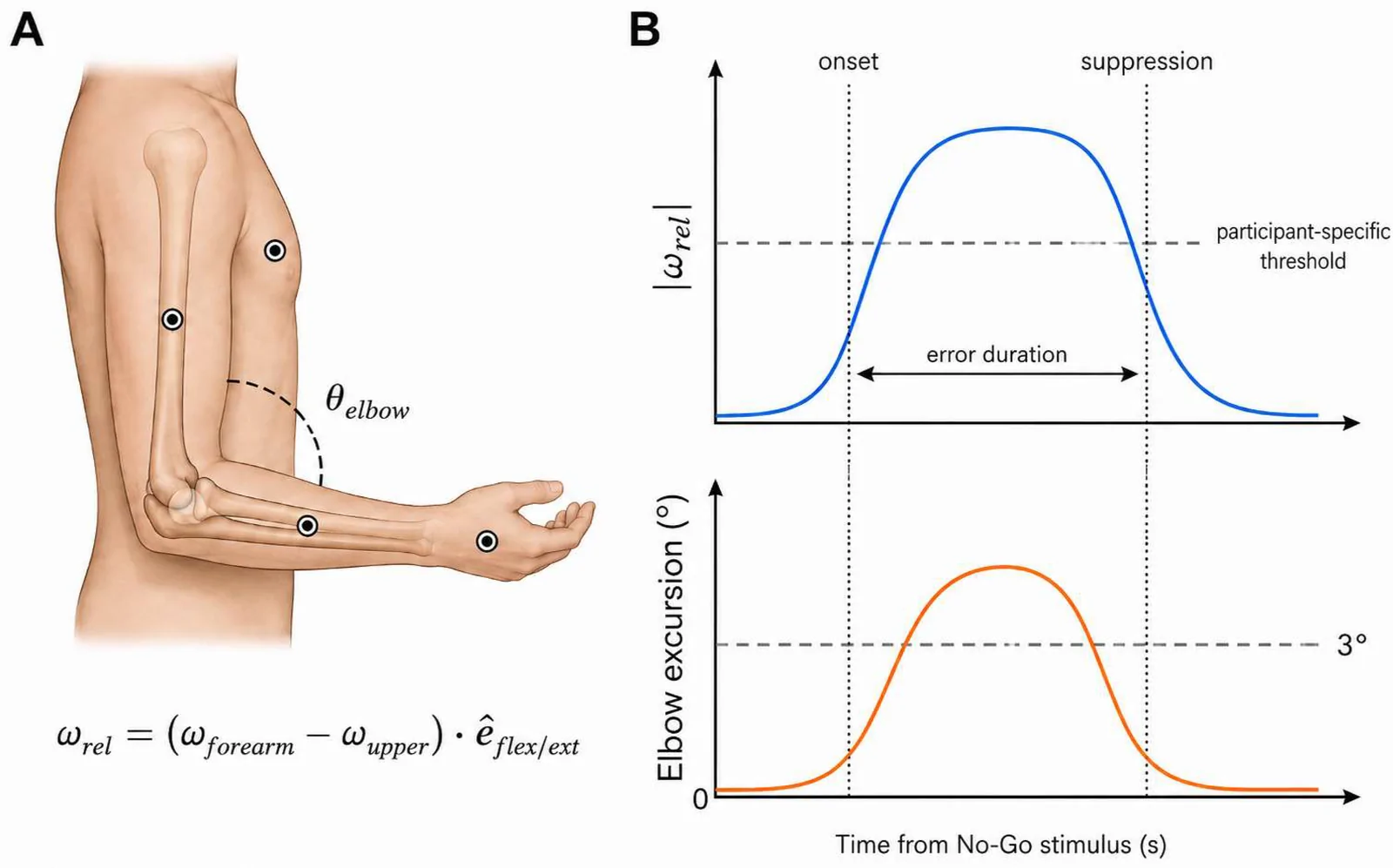
Schematic representation of elbow kinematics and commission-error detection. (**A**) Four IMUs were positioned on the sternum, upper arm, forearm, and dorsum of the hand. Elbow flexion–extension kinematics were derived from the upper-arm and forearm sensors; relative elbow angular velocity was obtained from the difference between segment angular velocities projected onto the calibrated flexion–extension axis, while elbow angle was derived from their relative orientation. (**B**) A commission error was identified when absolute relative elbow angular velocity exceeded the participant-specific threshold for at least 50 ms and was accompanied by an elbow excursion of at least 3°. Error duration was defined as the interval between movement onset and movement suppression. Curves are schematic and shown for illustrative purposes only.

Raw inertial data were processed to identify task-relevant elbow flexion–extension movements during No-Go trials. Sensor-to-segment alignment was established using a standardized static posture and functional elbow flexion–extension movements were performed before the experimental task. Upper-arm and forearm angular velocities were transformed into a common reference frame. Relative elbow angular velocity was calculated by subtracting upper-arm angular velocity from forearm angular velocity and projecting the resulting vector onto the calibrated flexion–extension axis. Elbow flexion–extension angle was obtained from the relative orientation of the forearm and upper-arm sensors (Figure 2A). The resulting kinematic signals were low-pass filtered using a fourth-order, zero-phase Butterworth filter with a cutoff frequency of 10 Hz.

For each No-Go trial, movement detection was performed within the response interval extending from stimulus onset to immediately before the subsequent stimulus. A commission error was identified when the absolute task-relevant relative elbow angular velocity exceeded a participant-specific threshold for at least 50 ms and was accompanied by an absolute elbow flexion–extension excursion of at least 3°. The participant-specific threshold was defined as the mean absolute relative elbow angular velocity recorded during the resting period plus three standard deviations. The combined velocity and angular-displacement criteria were used to distinguish genuine task-related responses from sensor noise, minor postural adjustments, and isolated movements of the hand, wrist, shoulder, or trunk.

Movement onset was defined as the first sample of a sustained threshold crossing satisfying both the velocity and angular-excursion criteria. Movement suppression was defined as the first subsequent time point at which the absolute relative elbow angular velocity returned below the participant-specific threshold and remained below it for at least 100 ms. Error duration was calculated as the interval, in seconds, between movement onset and movement suppression (Figure 2B). Error onset was searched within the No-Go response interval, but duration estimation was not mechanically set to zero or capped solely because the next stimulus occurred. Movements involving the trunk, shoulder, wrist, or hand without a corresponding task-relevant change in relative elbow flexion–extension kinematics were not classified as commission errors.

The error-detection procedure was validated in 320 No-Go trials sampled from the complete set of 6360 No-Go trials. Automated IMU classification was used only for stratification: 160 trials were drawn from the 3185 trials automatically classified as commission errors and 160 from the 3175 trials automatically classified as no-error trials (sampling fractions: 5.024% and 5.039%, respectively). Within each category, sampling was proportional across the 12 stimulus rate × block strata and was performed without replacement. Two independent assessors, blinded to the automated classification, reviewed the synchronized kinematic traces and stimulus markers and independently classified each selected trial as containing or not containing a commission error. Disagreements were resolved by consensus, which served as the reference standard. Sensitivity represented the proportion of manually confirmed commission errors detected by the automated procedure, whereas specificity represented the proportion of manually confirmed no-error trials correctly classified. No sampling weights were applied when calculating overall agreement, sensitivity, specificity, or Cohen’s κ.

Following error detection, the trial-level dataset contained the classification of each No-Go trial and, when a commission error occurred, the corresponding erroneous-movement duration. For each 50-stimulus series, the dataset contained either an error-duration value or a blank entry for each of the 10 No-Go trials. Blank entries indicated the absence of a commission error rather than missing data.

Three outcomes were calculated from the trial-level data. Commission-error probability was calculated as the number of commission errors divided by the 10 No-Go trials included in each series. Conditional error duration was calculated as the mean duration of erroneous movements within a series and was therefore defined only for series containing at least one commission error. This outcome described the persistence of an erroneous movement after action restraint had failed.

The integrated error index combined error frequency and erroneous-movement duration. The exponent of 1.5 was used as a pragmatic weighting to assign progressively greater influence to series characterized by a high number of commission errors while retaining information on erroneous-movement duration. The index was calculated as follows:

Integrated error index = (number of commission errors)^1.5^ × mean error duration

For series containing no commission errors, the integrated error index was set to 0. Higher values indicated poorer inhibitory performance. Application of these calculations yielded 636 series-level observations corresponding to 53 participants, four stimulus rates, and three experimental blocks, for a total of 6360 No-Go trials. The integrated error index was interpreted as a measure of total error burden rather than as a process-pure estimate of a single inhibitory mechanism. Because the exponent of 1.5 represents a pragmatic weighting rather than an externally validated parameter, interpretive priority was assigned to the directly observed components: commission-error occurrence first, conditional error duration as a complementary outcome, and the integrated error index as a secondary composite measure of total error burden.

### 2.5. Statistical Analysis

Descriptive analyses used participant-level means. Integrated error index analyses were initially performed using Friedman tests and Holm-adjusted Wilcoxon signed-rank comparisons. The main analysis used participant fixed-effects regression with standard errors clustered by participants. This approach used all repeated series-level observations, controlled for all stable participant characteristics, and permitted direct estimation of stimulus-rate-by-block effects within individuals.

Commission-error occurrence was analyzed at the trial level using a binomial generalized estimating equation (GEE) with a logit link, participant as the clustering unit, an exchangeable working correlation structure, and robust sandwich covariance. Stimulus rate, block, and their interaction were entered as categorical predictors. Conditional error duration was analyzed only in series with at least one commission error and was log-transformed before modeling. Because of its skewed distribution, the integrated error index was analyzed as log(1 + index) in the primary omnibus model. Raw-scale participant fixed-effects models were additionally used as sensitivity analyses and to estimate the Block-3-minus-Block-1 contrasts, allowing absolute changes to be expressed in the original index units. Stimulus rate and block were treated as categorical predictors in the omnibus models. When a stimulus-rate-by-block interaction was detected, follow-up tests used model-based Block-1-to-Block-3 contrasts and direct difference-in-differences relative to 40 bpm, estimated from participant fixed-effects models with participant-clustered standard errors. Holm correction was applied across the three direct contrasts comparing 50, 60, and 70 bpm with 40 bpm.

For temperament moderation analyses, stimulus rate was expressed in 10 bpm units and centered at the sample mean, while block was centered at Block 2. A quadratic stimulus-rate term and the stimulus-rate-by-block interaction were retained in the model. Cross-level stimulus-rate-by-temperament and block-by-temperament interaction terms were then entered simultaneously for the five standardized temperament dimensions. Benjamini–Hochberg false-discovery-rate correction [24] was applied across the 10 stimulus-rate-by-temperament and block-by-temperament interaction tests within each outcome. Correction across all 30 temperament interaction tests was also examined as a global sensitivity analysis.

A participant-level sensitivity analysis regressed Block-3-minus-Block-1 change on all five standardized temperament scores using heteroskedasticity-robust standard errors (HC3); negative values indicate a reduction in the integrated error index. Because Block was treated as a categorical factor in the main task models, the Block-3-minus-Block-1 contrast did not assume a monotonic trajectory through Block 2. Participant-level block-profile shapes were additionally examined descriptively. A parallel model examined the total integrated error score. As a sample-size sensitivity benchmark for the five-predictor participant-level model (n = 53; residual df = 47), a two-sided α of 0.05 provided approximately 80% power for a partial correlation of |r| = 0.39; smaller trait associations were therefore expected to be estimated with limited precision. Exploratory associations between participant-level variability in erroneous-movement duration and affective temperament were examined using Spearman correlations. Variability was quantified across each participant’s commission-error trials using both the standard deviation (SD) and coefficient of variation (CV), with FDR correction applied across the 10 temperament–variability tests. The revised analyses were performed in Python 3.13.5 using pandas 2.2.3, NumPy 2.3.5, statsmodels 0.14.6, and SciPy 1.17.0. Pandas and NumPy were used for data management, transformations, and numerical calculations; statsmodels was used for fixed-effects regression models, trial-level binomial GEE, robust covariance estimation, model contrasts, and Wald hypothesis testing; and SciPy was used for nonparametric and correlation analyses. The moderation analyses were not preregistered and were therefore treated as exploratory.

## 3. Results

### 3.1. Sample Characteristics and Outcome Data

The study included 53 physically active participants (27 males and 26 females), comprising three adolescents (<18 years) and 50 adults. Mean age was 24.52 ± 3.57 years (range: 15.66–30.54 years); mean body mass was 66.12 ± 11.34 kg (range: 40–96 kg), and mean height was 171.21 ± 8.90 cm (range: 154–195 cm). The most frequently reported activities were gym-based exercise (n = 27), swimming (n = 21), soccer (n = 19), volleyball (n = 9), dance (n = 7), tennis (n = 6), and basketball (n = 5); participants could report more than one activity. The mean current weekly training frequency was 5.72 ± 1.55 sessions per week (median, 6; IQR, 5–7; range, 3–9). The full sport/activity distribution and reported years of experience are provided in Supplementary Table S7. All participants met the eligibility criterion of structured training at least three times per week during the preceding six months. Participant characteristics and affective-temperament scores are summarized in Table 1. Internal consistency was high across the five Brief TEMPS-M subscales in the present sample: depressive, α = 0.940; cyclothymic, α = 0.960; hyperthymic, α = 0.889; irritable, α = 0.972; and anxious, α = 0.951.

Across the 636 series-level observations, 3185 commission errors were identified among 6360 No-Go trials, corresponding to an overall commission-error rate of 50.1%. Twenty-three series contained no commission errors (3.6%); therefore, conditional error duration was available for 613 series. Duration-window checks showed that no error duration exceeded the nominal inter-stimulus interval at 40, 50, or 60 bpm. At 70 bpm, five of 870 error trials (0.6%) exceeded the nominal 0.86 s interval, indicating that duration was not simply capped at the next stimulus, although overlap with the following stimulus was possible in a very small number of fastest-rate errors.

### 3.2. Validation of the IMU-Based Error-Detection Procedure

The validation subset comprised 320 No-Go trials contributed by all 53 participants. Before consensus, the two independent assessors agreed on 304 of 320 trials (95.0% observed agreement); the remaining 16 disagreements were resolved by consensus. Compared with the resulting consensus classification, the automated IMU-based procedure correctly classified 301 of 320 trials, corresponding to an overall agreement of 94.1%. Sensitivity was 93.8%; specificity was 94.4%, and Cohen’s κ was 0.88, indicating excellent agreement beyond chance.

### 3.3. Stimulus Rate, Block, and Their Interaction

The participant-averaged integrated error index increased with stimulus rate, from 2.35 ± 1.80 at 40 bpm to 3.07 ± 2.09 at 50 bpm and 5.39 ± 3.06 at 60 bpm, remaining elevated at 70 bpm (5.08 ± 3.01). The Friedman test showed a significant effect of stimulus rate, where χ^2^(3) = 73.42, *p* < 0.001, and Kendall’s W = 0.46. Across blocks, the integrated error index decreased from 4.51 ± 2.29 in Block 1 to 3.93 ± 2.33 in Block 2 and 3.49 ± 2.20 in Block 3, where χ^2^(2) = 15.21, *p* < 0.001, and Kendall’s W = 0.14.

**Figure 3:**
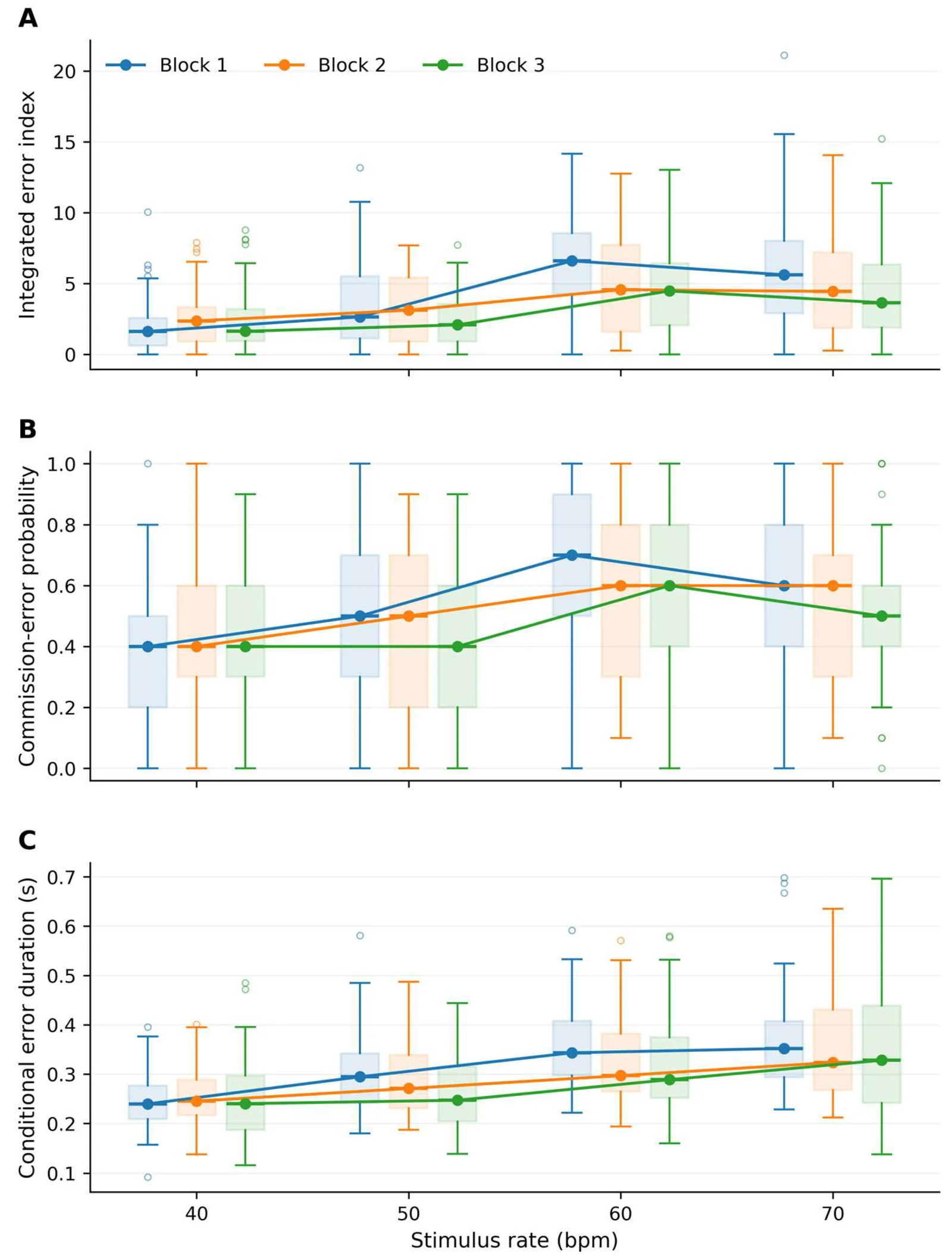
Distribution of inhibitory-performance outcomes across stimulus rates and experimental blocks. Boxplots show the integrated error index (**A**), commission-error probability (**B**), and conditional error duration (**C**) for Blocks 1–3 at each stimulus rate. Boxes represent the interquartile range; horizontal lines indicate medians; whiskers extend to 1.5 times the interquartile range, and points beyond the whiskers represent outliers. Lines and markers connect the medians within each experimental block. Lower values indicate lower error burden.

The trial-level binomial GEE showed a significant stimulus-rate-by-block interaction for commission-error occurrence (Wald χ^2^(6) = 25.96, *p* < 0.001). The main effect of stimulus rate was significant (χ^2^(3) = 63.72, *p* < 0.001), whereas the omnibus main effect of block was not (χ^2^(2) = 4.41, *p* = 0.110; Supplementary Table S3). Significant stimulus-rate-by-block interactions were also observed for conditional error duration and the integrated error index in the participant fixed-effects models (Table 2; Figure 3). For the integrated index, the log(1 + index) omnibus interaction was significant (χ^2^(6) = 30.40, *p* < 0.001), and the raw-scale fixed-effects sensitivity analysis independently confirmed the interaction (χ^2^(6) = 37.92, *p* < 0.001). Direct raw-scale contrasts confirmed that Block-1-to-Block-3 change in the integrated index differed from the 40 bpm reference at each faster rate. Estimated B3−B1 change was +0.32 points at 40 bpm, compared with −1.19 points at 50 bpm, −1.66 points at 60 bpm, and −1.55 points at 70 bpm. The corresponding difference-in-differences relative to 40 bpm were −1.51, −1.98, and −1.87 points, respectively (all Holm-adjusted *p* < 0.002; Table 3). The same interaction remained significant when the integrated index was recalculated as total erroneous-movement time (exponent 1.0; Wald χ^2^(6) = 33.04, *p* < 0.001) or with stronger frequency weighting (exponent 2.0; Wald χ^2^(6) = 28.29, *p* < 0.001). Because rate order was randomized within each block, systematic serial-position bias is unlikely. Aggregated across stimulus rates, 23/53 participants showed a monotonic decrease in the integrated error index, 6/53 a monotonic increase, 11/53 a U-shaped integrated error profile with the lowest error burden at Block 2, and 13/53 an inverted-U profile with the highest error burden at Block 2. Overall, 38/53 participants had a lower integrated error index at Block 3 than at Block 1 (Supplementary Table S4).

### 3.4. Affective Temperament and Performance Dynamics

The five Brief TEMPS-M dimensions showed intercorrelations ranging from −0.174 to 0.593 in the present sample (Supplementary Table S5). No temperament-by-stimulus-rate interaction survived FDR correction for any outcome; the full set of cross-level temperament interaction tests is reported in Supplementary Table S1. In the integrated error index model, higher irritable temperament was associated with a steeper decline in the integrated error index across blocks, indicating a larger within-session reduction in the index (interaction estimate = −0.073 log-index units per block per SD, *p* = 0.0049; FDR *p* = 0.0489 across the 10 integrated index interaction tests). The corresponding association was directionally similar for commission-error probability, with a reduction of 2.1 percentage points per block per SD increase in irritable temperament (*p* = 0.014), but it did not survive FDR correction. The association with conditional error duration was weaker and not statistically significant (*p* = 0.082). When FDR correction was applied globally across all 30 temperament interaction tests, the irritable-temperament-by-block association did not remain significant (FDR *p* = 0.147).

**Figure 4:**
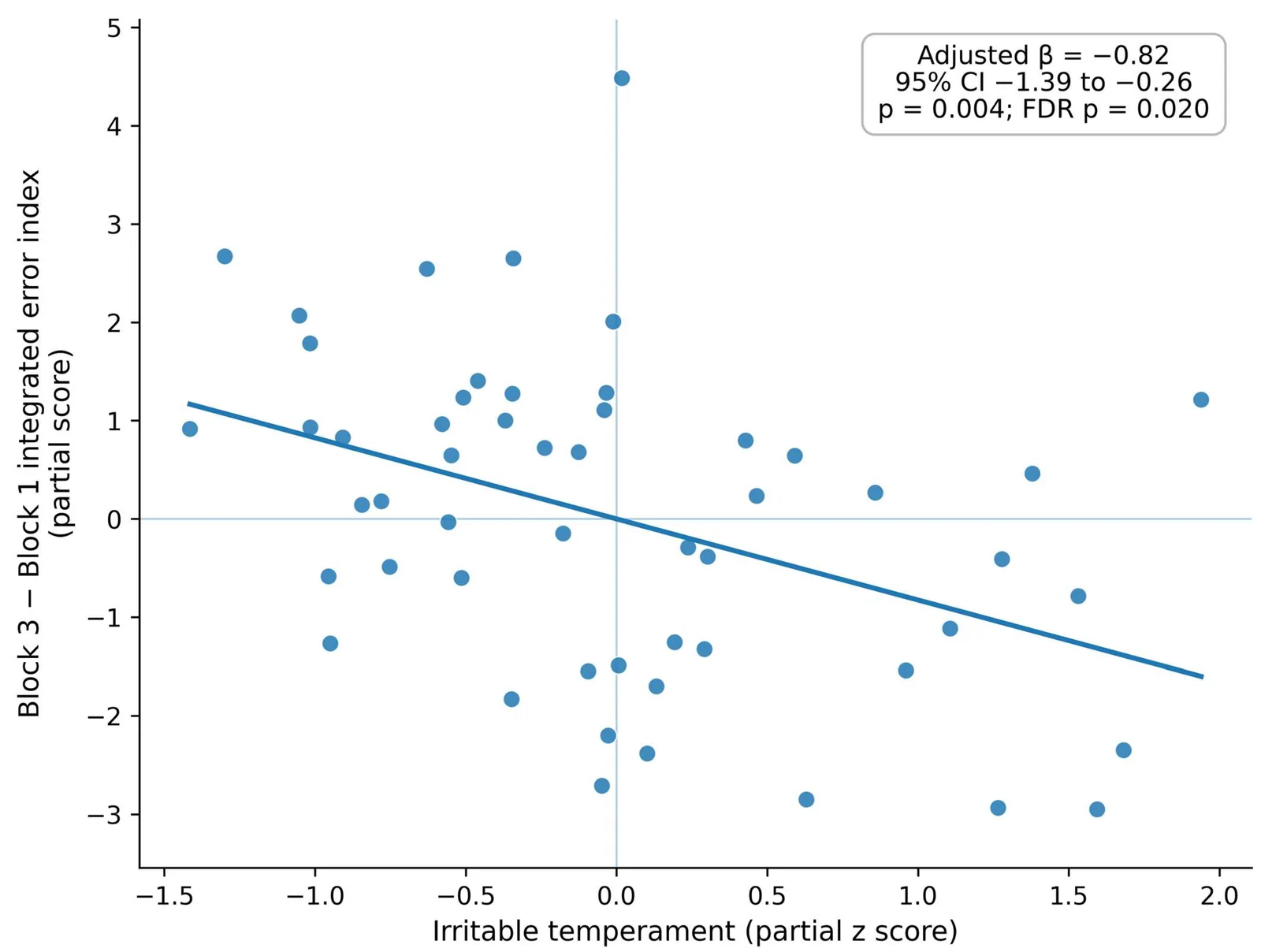
Partial association between irritable temperament and Block-3-minus-Block-1 change in the integrated error index. Both variables are residualized for depressive, cyclothymic, hyperthymic, and anxious temperament scores for visualization. Negative values on the y-axis indicate a larger reduction in error burden from Block 1 to Block 3. The annotation reports the corresponding coefficient from the full HC3-robust multivariable model.

The secondary participant-level sensitivity analysis showed a similar exploratory pattern, with complete adjusted models reported in Supplementary Table S2. After adjustment for depressive, cyclothymic, hyperthymic, and anxious temperament scores, each 1-SD increase in irritable temperament was associated with a 0.82-point more negative Block-3-minus-Block-1 change in the integrated error index (β = −0.82, 95% CI −1.39 to −0.26, *p* = 0.004; FDR *p* = 0.020 across the five traits; Figure 4). Negative change denotes a reduction in the integrated error index. This analysis used the same sample and should be interpreted as a participant-level sensitivity analysis rather than as independent confirmation of the moderation result. Hyperthymic temperament showed a suggestive association with a lower total integrated error score (β = −8.58 points per SD, 95% CI −15.40 to −1.76, *p* = 0.014), but this association did not survive FDR correction (FDR *p* = 0.068). In an additional exploratory analysis, irritable temperament was nominally associated with greater variability in erroneous-movement duration, both for the SD (Spearman ρ = 0.340, *p* = 0.013) and the coefficient of variation (ρ = 0.273, *p* = 0.048). However, neither association survived FDR correction across the 10 temperament–variability tests (adjusted *p* = 0.127 and 0.239, respectively; Supplementary Table S6). Selected findings from these models are summarized in Table 4.

## 4. Discussion

This study examined two related questions. The primary objective was to characterize within-session No-Go motor performance across stimulus-rate conditions. The secondary objective was to explore whether affective temperament contributes to these within-session performance dynamics. Physically active participants, predominantly young adults, completed repeated blocks of an elbow-based Go/No-Go task at four randomized stimulus rates. The main finding was that within-session change in No-Go motor performance differed across stimulus-rate conditions: although overall error burden was lower at 40 bpm, little change occurred across blocks at this lowest stimulus rate, whereas reductions emerged at 50, 60, and 70 bpm. These reductions involved both commission-error occurrence and erroneous-movement duration. In contrast, temperament explained comparatively little of the overall pattern, although irritable temperament showed an exploratory association with Block-1-to-Block-3 change.

The observed rate-associated pattern should therefore be interpreted descriptively. Randomization of rate order within each block reduces the likelihood of a systematic serial-position effect, but it does not resolve the confounding between stimulus rate and sequence content. Each rate retained its own predefined stimulus sequence, including fixed No-Go positions, across all three blocks and all participants. Consequently, differences between rate conditions and differences in their Block-1-to-Block-3 trajectories may reflect stimulus timing, sequence-specific familiarization, or a combination of both. The present data establish that within-session No-Go motor performance differed across the rate-associated conditions; they do not isolate temporal demand as the causal source of those differences.

The findings remain compatible with the broader view that inhibitory performance can vary with task structure and context, but mechanistic interpretation should remain cautious. This is consistent with evidence that inhibitory-control performance varies according to task structure, context, and the specific inhibitory process required [4,16]. At slower rates, participants may have had sufficient time to identify the stimulus, select the appropriate response, and suppress the No-Go response, leaving little room for measurable within-session change. This interpretation is consistent with previous Go/No-Go studies showing that temporal constraints strongly influence inhibitory performance, with longer inter-stimulus intervals associated with greater accuracy and fewer commission errors [25,26]. Moreover, short-term practice effects have been shown to depend on task demands, with smaller behavioral changes under low-demand conditions [13]. At faster rates, the task likely increased the need for rapid stimulus classification, response-threshold adjustment, and online correction of inappropriate movements. Under these conditions, within-session change was more evident. The present outcomes characterize No-Go performance specifically; complementary Go-trial measures would be useful in future work to determine how much of the within-session change reflects inhibitory adjustment versus a broader shift in response strategy. This distinction is consistent with broader accounts of motor inhibition in which reactive stopping operates alongside proactive adjustments of motor preparation and response strategy [27]. The decomposed outcomes clarify the behavioral pattern underlying this within-session change.

Commission-error occurrence directly indexes failure to withhold the instructed response and therefore has primary interpretive priority. Conditional error duration reflects the persistence of the erroneous movement after restraint has failed; it is informative about online correction but should not be considered a process-pure measure of action cancelation [4,16]. The integrated error index summarizes frequency and duration but uses a pragmatic 1.5 exponent that has not been externally validated. For this reason, it is secondary to the component outcomes. The fact that the rate-by-block interaction remained significant with frequency exponents of 1.0, 1.5, and 2.0 supports robustness of the qualitative pattern, but it does not validate 1.5 as a uniquely correct weighting.

The present findings indicate within-session changes in No-Go motor performance under different stimulus-rate conditions. Several mechanisms could potentially contribute to these within-session changes, including changes in stimulus classification, anticipation of No-Go events, response-threshold setting, or correction of escaped actions. Previous Go/No-Go studies have reported reductions in false alarms and changes in neural responses after short practice [12,13]. Moreover, response-inhibition training has been linked to changes in motor-cortical inhibitory physiology, even when average behavioral changes are modest [14]. Although the present study did not measure neural activity, the observed behavioral pattern does not identify the mechanism responsible. Changes in response threshold, anticipation, online correction, or other strategic adjustments could all contribute.

The temperament results address the exploratory individual-difference objective and require especially cautious interpretation given the modest sample size and the simultaneous evaluation of multiple correlated traits. Affective temperament may plausibly influence cognitive-motor inhibition because emotional reactivity, arousal, attentional control, and self-regulation are all relevant when individuals must suppress or correct actions under time pressure [17,18,19,20,21]. In the present study, irritable temperament was associated with a more negative Block-3-minus-Block-1 change in the participant-level model and with a steeper block-related decline in the integrated error index. One possible interpretation is that individuals with higher irritable temperament showed a larger Block-1-to-Block-3 reduction once task contingencies became familiar. However, alternative explanations are also plausible, including greater initial error burden, regression toward the mean, residual overlap among temperament dimensions, and sampling variability. Therefore, this result should be considered exploratory and hypothesis-generating rather than evidence that irritable temperament enhances inhibitory learning.

Hyperthymic temperament showed a suggestive association with lower overall error burden, broadly consistent with the idea that hyperthymic traits may relate to energy, activation, or resilience [28]. However, this association did not remain significant after correction across temperament dimensions. More generally, neither hyperthymic temperament nor the other temperament dimensions robustly moderated sensitivity to increasing stimulus rate. The rate- and block-associated performance pattern was therefore more prominent than temperament-related variation. In practical terms, affective temperament contributed comparatively little to the main No-Go performance pattern.

Several limitations should be acknowledged, although they do not undermine the main behavioral finding. First, the study used a within-session design; therefore, the observed changes across blocks should be interpreted as within-session changes in No-Go performance rather than as stable learning. Second, although the order of the four rate conditions was randomized within each block, each rate retained the same predefined stimulus sequence across blocks. Therefore, repeated exposure to rate-specific No-Go positions may have contributed to sequence-specific familiarization, and this cannot be fully separated from more general within-session changes in task performance. Other time-dependent factors such as fatigue, arousal, motivation, or strategic adjustment may also have contributed to the observed pattern. Third, Go-trial accuracy and response-time measures were not included in the present analysis; therefore, the observed within-session changes cannot be distinguished from an overall slowing or a broader shift in response strategy and should be interpreted specifically as changes in No-Go motor performance rather than as a process-pure measure of a specific inhibitory mechanism. Fourth, duration-based outcomes are partly linked to the temporal structure of the task; however, only five error trials exceeded the nominal inter-stimulus interval, and the main interaction remained evident for commission-error probability as well as duration-based outcomes. Fifth, the integrated error index uses a pragmatic weighting and lacks external validation, so the directly observed components should carry greater interpretive weight. However, sensitivity analyses using alternative frequency weightings and the raw-scale model supported the same qualitative rate-by-block pattern. Finally, the sample was modest and composed predominantly of physically active young adults with heterogeneous sport backgrounds, and the temperament analyses were exploratory and should be confirmed in larger preregistered studies.

Despite these limitations, the study has several strengths. It included all available No-Go trials, verified the accuracy and internal consistency of the outcome calculations, and applied within-participant models rather than relying only on aggregated totals. Future studies should use independently randomized or counterbalanced stimulus sequences across rate conditions and examine whether the present rate-associated within-session pattern replicates across different cognitive-motor inhibition tasks, sport profiles, and levels of physical activity. Larger samples would also allow more precise testing of affective temperament as a moderator of within-session No-Go motor performance change.

Overall, No-Go motor performance changed across blocks and differed across stimulus-rate conditions. Because stimulus rate and sequence content were confounded, the present data do not establish that temporal demand itself produced these differences. Affective temperament was relevant only as an exploratory individual-difference factor, with irritable temperament showing the clearest but preliminary association with Block-1-to-Block-3 change. The central message is therefore descriptive: within-session No-Go motor performance showed a pattern that differed across rate-associated conditions, while temperament-related modulation remains preliminary.

## 5. Conclusions

In this cognitive-motor Go/No-Go task, within-session No-Go motor performance differed across stimulus-rate conditions. Block-1-to-Block-3 reductions emerged at 50–70 bpm but not at 40 bpm and were evident in both commission-error occurrence and erroneous-movement duration. Because each stimulus rate was paired with a fixed repeated stimulus sequence, these rate-associated differences cannot be attributed uniquely to temporal demand and may partly reflect sequence-specific familiarization. These findings support short-term, context-sensitive performance change while remaining cautious about attributing the pattern to an isolated inhibitory mechanism. Affective temperament explained comparatively little of these dynamics, although irritable temperament showed an exploratory association with Block-1-to-Block-3 change. Future preregistered studies should test whether these rate-associated differences replicate across different cognitive-motor inhibition tasks, sport profiles, and levels of physical activity.

## Figures and Tables

**Table 1:** Sample characteristics and affective-temperament scores.

Characteristic	Value	Range
Participants	53	—
Sex	27 males; 26 females	—
Age, years	24.52 ± 3.57	15.66–30.54
Body mass, kg	66.12 ± 11.34	40–96
Height, cm	171.21 ± 8.90	154–195
Weekly training frequency, sessions/week	5.72 ± 1.55	3–9
Depressive temperament	17.26 ± 4.98	9–30
Cyclothymic temperament	17.04 ± 6.13	7–29
Hyperthymic temperament	22.68 ± 3.69	15–32
Irritable temperament	14.42 ± 6.46	7–32
Anxious temperament	15.55 ± 5.32	7–31

Note. Values are reported as mean ± standard deviation unless otherwise indicated. Range indicates the observed minimum and maximum values.

**Table 2:** Omnibus stimulus rate, block, and interaction tests for task outcomes.

Outcome	Stimulus Rate χ^2^ (df), *p*	Block χ^2^ (df), *p*	Stimulus Rate × Block χ^2^ (df), *p*
Commission-error occurrence ^1^	63.72 (3), <0.001	4.41 (2), 0.110	25.96 (6), <0.001
Conditional error duration ^2^	77.03 (3), <0.001	12.45 (2), 0.002	14.96 (6), 0.021
Integrated error index ^3^	115.48 (3), <0.001	15.12 (2), <0.001	30.40 (6), <0.001

Note. ^1^ Commission-error occurrence was analyzed at the trial level using a binomial GEE with logit link, exchangeable working correlation, participant clustering, and robust sandwich covariance. ^2^ Conditional error duration was analyzed using a participant fixed-effects model fitted to log duration among series containing at least one error. ^3^ The integrated error index was analyzed using a participant fixed-effects model fitted to log(1 + index).

**Table 3:** Model-based Block-3-minus-Block-1 change in the integrated error index and direct contrasts against 40 bpm.

Rate	B3 − B1 Change	DiD vs. 40 bpm	DiD 95% CI	Holm *p*
40 bpm	0.32 (−0.31 to 0.95)	Reference	--	--
50 bpm	−1.19 (−1.95 to −0.44)	−1.51	−2.29 to −0.74	<0.001
60 bpm	−1.66 (−2.37 to −0.96)	−1.98	−2.90 to −1.07	<0.001
70 bpm	−1.55 (−2.63 to −0.48)	−1.87	−3.02 to −0.72	0.001

Note. B3 − B1 changes and difference-in-differences are raw-scale participant fixed-effects estimates used to express absolute change in the original index units. The primary omnibus composite model used log(1 + index) because of skewness, and the raw-scale interaction was also significant. Negative values indicate a reduction in the integrated error index. Difference-in-differences = (B3 − B1)rate − (B3 − B1)40 bpm; Holm *p*-values are adjusted across the three contrasts.

**Table 4:** Main exploratory temperament findings from adjusted moderation and participant-level sensitivity analyses.

Predictor	Outcome	Estimate	Robust SE	*p*	FDR *p*
Irritable × block	Integrated index	−0.073	0.026	0.0049	0.0489 ^1^
Irritable × block	Commission probability	−0.0209	0.0085	0.014	0.145 ^2^
Irritable × block	Conditional duration	−0.031	0.018	0.082	0.429 ^2^
Irritable temperament	Block-3-minus-Block-1 change	−0.82	0.29	0.004	0.020 ^3^
Hyperthymic temperament	Total integrated score	−8.58	3.48	0.014	0.068 ^3^

Note. ^1^ FDR across the 10 stimulus-rate- and block-moderation terms for the integrated index. ^2^ FDR across the 10 interactions for the corresponding decomposed outcome. ^3^ FDR across five traits in the participant-level model. Participant-level change is expressed as Block-3-minus-Block-1, so negative values indicate a reduction in the integrated error index.

## Data Availability

The anonymized data and reproducibility materials supporting the analyses are provided with the revised submission, including participant-, series-, and No-Go-trial-level datasets, analysis scripts, reviewer-requested sensitivity analyses, non-identifying demographic and sport-history summaries, exact rate-specific stimulus-sequence definitions, and validation-summary information.

## Supplementary Materials

The following supporting information can be downloaded at https://www.mdpi.com/article/10.3390/life16081368/s1, Table S1: Cross-level temperament interactions in the joint fixed-effects models; Table S2: Participant-level adjusted temperament models; Table S3: Trial-level binomial GEE omnibus tests for commission-error occurrence; Table S4: Participant-level block-profile classifications for the integrated error index; Table S5: Pearson intercorrelations among Brief TEMPS-M dimensions; Table S6: Spearman associations of erroneous-movement-duration variability with affective temperament; Table S7: Reported sport and exercise histories and overall weekly training exposure; Table S8: Predefined rate-specific stimulus-sequence structure and No-Go positions.
