# Supplementary material for: Rate-Associated Differences in Within-Session Motor-Inhibitory Performance: Exploratory Role of Affective Temperament
Source: Life (Basel). 2026 Aug 19;16(8):1368. doi: 10.3390/life16081368 (PMC13514360; doi:10.3390/life16081368)
Supplement: Supplementary file 1 [file life-16-01368-s001.zip › life-4479831-supplementary.pdf]

Rate-Associated Differences in Within-Session Motor-Inhibitory Performance: Exploratory Role of Affective Temperament

Supplementary Tables

Table S1. Cross-level temperament interactions in the joint fixed-effects models.

Outcome	Domain	Trait	B	SE	p	Outcome FDR	Global FDR
Commission-error probability	Stimulus rate	Depressive	0.010	0.012	.367	.719	.836
Commission-error probability	Stimulus rate	Cyclothymic	0.006	0.010	.525	.719	.836
Commission-error probability	Stimulus rate	Hyperthymic	-0.009	0.007	.228	.570	.710
Commission-error probability	Stimulus rate	Irritable	-0.010	0.007	.198	.570	.710
Commission-error probability	Stimulus rate	Anxious	-0.004	0.009	.653	.726	.852
Commission-error probability	Block	Depressive	0.002	0.011	.874	.874	.914
Commission-error probability	Block	Cyclothymic	-0.010	0.014	.471	.719	.836
Commission-error probability	Block	Hyperthymic	-0.005	0.009	.575	.719	.852
Commission-error probability	Block	Irritable	-0.021	0.009	.014	.145	.217
Commission-error probability	Block	Anxious	0.012	0.009	.165	.570	.709
Conditional error duration	Stimulus rate	Depressive	0.023	0.015	.129	.429	.644
Conditional error duration	Stimulus rate	Cyclothymic	-0.003	0.021	.878	.931	.914
Conditional error duration	Stimulus rate	Hyperthymic	-0.002	0.018	.931	.931	.931
Conditional error duration	Stimulus rate	Irritable	0.008	0.018	.652	.931	.852
Conditional error duration	Stimulus rate	Anxious	0.006	0.022	.794	.931	.914
Conditional error duration	Block	Depressive	0.029	0.018	.101	.429	.603
Conditional error duration	Block	Cyclothymic	-0.002	0.017	.883	.931	.914
Conditional error duration	Block	Hyperthymic	-0.009	0.013	.498	.931	.836
Conditional error duration	Block	Irritable	-0.031	0.018	.082	.429	.603
Conditional error duration	Block	Anxious	0.005	0.016	.734	.931	.881
Integrated error index	Stimulus rate	Depressive	0.053	0.029	.066	.332	.603
Integrated error index	Stimulus rate	Cyclothymic	0.018	0.029	.529	.662	.836
Integrated error index	Stimulus rate	Hyperthymic	-0.011	0.021	.603	.670	.852
Integrated error index	Stimulus rate	Irritable	-0.020	0.023	.383	.662	.836
Integrated error index	Stimulus rate	Anxious	-0.009	0.027	.721	.721	.881
Integrated error index	Block	Depressive	0.029	0.032	.379	.662	.836
Integrated error index	Block	Cyclothymic	-0.024	0.034	.477	.662	.836
Integrated error index	Block	Hyperthymic	-0.019	0.026	.467	.662	.836
Integrated error index	Block	Irritable	-0.073	0.026	.0049	.0489	.147
Integrated error index	Block	Anxious	0.028	0.024	.237	.662	.710

Note. The outcome-specific FDR column corrects 10 interaction tests (five traits × two interaction domains: stimulus rate and block) within each outcome. The global FDR column corrects all 30 interaction tests across the three outcomes.

Table S2. Participant-level adjusted temperament models.

Outcome	Trait	B	HC3 SE	95% CI	p	FDR p
B3−B1 change	Depressive	0.36	0.30	-0.22 to 0.94	.224	.280
B3−B1 change	Cyclothymic	-0.47	0.31	-1.07 to 0.14	.133	.222
B3−B1 change	Hyperthymic	0.04	0.21	-0.36 to 0.45	.836	.836
B3−B1 change	Irritable	-0.82	0.29	-1.39 to -0.26	.004	.020
B3−B1 change	Anxious	0.49	0.24	0.02 to 0.97	.042	.105
Total integrated error score	Depressive	-3.08	3.93	-10.79 to 4.63	.433	.541
Total integrated error score	Cyclothymic	4.72	4.97	-5.01 to 14.46	.342	.541
Total integrated error score	Hyperthymic	-8.58	3.48	-15.40 to -1.76	.014	.068
Total integrated error score	Irritable	4.12	3.64	-3.02 to 11.25	.259	.541
Total integrated error score	Anxious	-0.42	4.60	-9.43 to 8.59	.927	.927

Note. Models used HC3-robust standard errors. Change is defined as the Block 3 mean minus the Block 1 mean; therefore, negative coefficients indicate a larger reduction in the integrated error index. This direction is consistent with Figure 4 and the block-related moderation results.

Table S3. Trial-level binomial GEE omnibus tests for commission-error occurrence.

Term	Wald χ^2	df	p
Stimulus rate	63.72	3	< .001
Block	4.41	2	.110
Stimulus rate × block	25.96	6	.000227

Note. The model included all 6,360 No-Go trials, with participant as the clustering unit (53 clusters), a logit link, an exchangeable working correlation structure, and robust sandwich covariance. The trial-level binomial GEE constituted the primary inferential analysis for commission-error occurrence.

Table S4. Participant-level block-profile classifications for the integrated error index.

Scope	Rate	Monotonic decrease	Monotonic increase	U-shape (B2 lowest)	Inverted-U (B2 highest)
Across-rate mean	All	23	6	11	13
Rate-specific	40 bpm	9	14	12	18
Rate-specific	50 bpm	18	7	13	15
Rate-specific	60 bpm	18	7	20	8
Rate-specific	70 bpm	15	5	17	16

Note. Across rates, 38 of 53 participants had a lower integrated error index in Block 3 than in Block 1. Block was modeled categorically in the main analyses; therefore, the Block 3-minus-Block 1 endpoint contrast did not impose monotonicity. U-shaped and inverted-U profiles explicitly show trajectories in which Block 2 differed from the endpoint trend.

Table S5. Pearson intercorrelations among Brief TEMPS-M dimensions (n = 53).

Trait	Depressive	Cyclothymic	Hyperthymic	Irritable	Anxious
Depressive	1.000	0.593	0.008	0.399	0.448
Cyclothymic	0.593	1.000	-0.067	0.552	0.530
Hyperthymic	0.008	-0.067	1.000	-0.083	-0.174
Irritable	0.399	0.552	-0.083	1.000	0.401
Anxious	0.448	0.530	-0.174	0.401	1.000

Note. Correlations ranged from -0.174 to 0.593. The five temperament dimensions were entered simultaneously in the adjusted models to account for their intercorrelations.

Table S6. Spearman associations of erroneous-movement-duration variability with affective temperament.

Metric	Trait	Spearman ρ	p	FDR p (metric)	FDR p (all 10)
SD	Depressive	0.200	.151	.377	.503
SD	Cyclothymic	0.133	.341	.568	.748
SD	Hyperthymic	0.062	.659	.659	.748
SD	Irritable	0.340	.013	.063	.127
SD	Anxious	0.094	.505	.631	.748
CV	Depressive	0.059	.673	.824	.748
CV	Cyclothymic	-0.093	.507	.824	.748
CV	Hyperthymic	0.031	.824	.824	.824
CV	Irritable	0.273	.048	.239	.239
CV	Anxious	0.076	.587	.824	.748

Note. Variability was calculated across each participant's commission-error trials. No variability-temperament association survived FDR correction. The nominal irritable-temperament associations were $\rho = .340$ for SD ($p = .013$; all-test FDR $p = .127$) and $\rho = .273$ for CV ($p = .048$; all-test FDR $p = .239$). SD = standard deviation; CV = coefficient of variation; FDR = false discovery rate.

Table S7. Reported sport and exercise histories and overall weekly training exposure.

Sport/activity	Participants, n	% of 52	Median experience, years	Range, years
Gym-based exercise	27	51.9	3.0	1.0-7.0
Swimming	21	40.4	5.0	1.0-16.0
Soccer	19	36.5	10.0	1.0-22.0
Volleyball	9	17.3	7.0	1.0-12.0
Dance	7	13.5	5.0	4.0-13.0
Tennis	6	11.5	3.5	3.0-16.0
Basketball	5	9.6	2.0	1.0-14.0
Artistic gymnastics	3	5.8	4.0	2.0-12.0
Boxing	3	5.8	2.0	0.25-3.0
Water polo	3	5.8	2.0	1.0-7.0
Karate	2	3.8	6.0	2.0-10.0
Kickboxing	2	3.8	4.0	2.0-6.0
Pole dance	2	3.8	1.92	1.83-2.0
Aikido	1	1.9	1.5	1.5-1.5
Archery	1	1.9	2.0	2.0-2.0
Artistic swimming	1	1.9	14.0	14.0-14.0
Athletics	1	1.9	2.0	2.0-2.0
Cheerleading	1	1.9	7.0	7.0-7.0
Cycling	1	1.9	1.0	1.0-1.0
Muay Thai	1	1.9	3.0	3.0-3.0
Rowing	1	1.9	0.5	0.5-0.5
Running	1	1.9	15.0	15.0-15.0
Skiing	1	1.9	16.0	16.0-16.0
Trekking	1	1.9	5.0	5.0-5.0

Note. Participants could report more than one activity, so counts do not sum to 52. Experience reflects the number of years reported for each activity. Current weekly training frequency was available for all 53 participants (mean ± SD, 5.72 ± 1.55 sessions/week; median, 6; IQR, 5–7; range, 3–9). Quantitative training duration in hours/week was not available.

Table S8. Predefined rate-specific stimulus-sequence structure and No-Go positions.

Rate	ISI, s	No-Go positions (1-50)	Sequence reuse	Rate order
40 bpm	1.500	3, 6, 18, 28, 30, 33, 38, 41, 47, 50	Same predefined sequence across all participants and Blocks 1-3	Randomized within each block
50 bpm	1.200	3, 11, 25, 27, 29, 31, 33, 35, 39, 47	Same predefined sequence across all participants and Blocks 1-3	Randomized within each block
60 bpm	1.000	3, 11, 21, 25, 29, 31, 34, 37, 43, 50	Same predefined sequence across all participants and Blocks 1-3	Randomized within each block
70 bpm	0.857	2, 6, 8, 14, 16, 19, 27, 41, 45, 48	Same predefined sequence across all participants and Blocks 1-3	Randomized within each block

Note. Each rate-specific sequence contained 20 elbow-flexion Go trials, 20 elbow-extension Go trials, and 10 No-Go trials. When No-Go trials were removed, Go trials alternated flexion and extension, and no sequence contained consecutive No-Go trials. No-Go positions are listed using 1-based trial numbering. The same predefined sequence for each rate was used for all participants and repeated across Blocks 1-3. Sequence content was fixed within stimulus rate and was not counterbalanced across participants; randomization concerned the order of the four rate conditions within each block. ISI = inter-stimulus interval.
